# Developing a patient journey map to improve care and experience in Chinese patients with hereditary angioedema^☆^

**DOI:** 10.1016/j.waojou.2026.101333

**Published:** 2026-01-30

**Authors:** Yin Wang, Yangxue Fu, Hao Chen, Jin Liu, Qingxiu Xu, Yaqi Yang, Si Zhang, Jing Cheng, Rongfei Zhu

**Affiliations:** a Department of Allergy, Tongji Hospital, Tongji Medical College, Huazhong University of Science and Technology, Wuhan, China; b Institute of Allergy and Clinical Immunology, Tongji Hospital, Tongji Medical College, Huazhong University of Science and Technology, Wuhan, China; c Department of Nursing, Tongji Hospital, Tongji Medical College, Huazhong University of Science and Technology, Wuhan, China

**Keywords:** Hereditary angioedema (HAE), Patient journey map (PJM), Patient-centered care, Quality of life (QoL)

## Abstract

**Background:**

Hereditary angioedema (HAE) is a rare disorder that imposes a substantial burden on patient health and quality of life. Although international studies have highlighted specific aspects of the HAE care continuum, comprehensive evidence on patient experiences in China remains scarce.

**Methods:**

We conducted a single-center, mixed-methods study to develop the first HAE Patient Journey Map (HAE-PJM) in China. Fifteen adult patients with HAE were recruited for structured interviews (July–November 2024), complemented by quantitative assessments using the Hospital Anxiety and Depression Scale (HADS) and the Angioedema Control Test (AECT). Stakeholder validation was obtained through expert–patient groups.

**Results:**

Patients experienced a mean diagnostic delay of 16.3 years, with 80% reporting misdiagnosis and high rates of inappropriate interventions, underscoring systemic deficiencies in early recognition. Pre-diagnosis, activity limitations, and anxiety were common; genetic and economic anxieties persisted even after treatment initiation despite improvements in HADS and AECT scores. Prophylactic regimens achieved superior disease control compared with on-demand therapy, yet access barriers—including cost, reimbursement, and drug availability—remained prevalent. Expert-patient group validation confirmed the relevance of a phased patient journey map and emphasized multidisciplinary roles, including physicians, nurses, navigators, and societal support systems.

**Conclusion:**

This study provides the first systematic HAE-PJM in China, integrating qualitative and quantitative evidence to delineate critical touchpoints and bottlenecks in HAE care. To address these identified challenges, we propose a “4T″ framework (Testing, Teaching, Therapeutic Monitoring, and Team working). These findings highlight urgent needs for standardized diagnostic pathways, integrated psychosocial and financial support, and access-oriented care models.

## Introduction

Hereditary angioedema (HAE) is a rare genetic disorder characterized by recurrent episodes and unpredictable attacks of subcutaneous and/or submucosal swelling. These attacks can be life-threatening and cause major discomfort, pain, fear, anxiety, and impaired quality of life for both patients and their caregivers.^1^ The estimated prevalence of HAE is 1:50,000 (range 1:10,000 to 1:100,000) in the general population.^2^ HAE results from dysregulated bradykinin-mediated vasodilation resulting from *SERPING1* mutations, which impair C1 inhibitor (C1INH), leading to a deficiency (HAE-C1INH-Type1, ∼85% of cases) or dysfunction HAE-C1INH-Type2, ∼15% of cases) of C1INH.^3^ In addition, a smaller subset of patients presents with HAE with normal C1INH (HAE-nC1INH). This form is most often associated with mutations in genes such as *F12*, *PLG*, *ANGPT1*, or *KNG1*, represents a distinct entity with clinical overlap with HAE-C1INH types.^4^^,^^5^ Unlike mast cell-mediated angioedema, bradykinin-induced vascular leakage in HAE is refractory to antihistamines or corticosteroids, highlighting the necessity for precise molecularly targeted interventions.^6^

HAE is increasingly recognized worldwide. Multiple studies have confirmed impairments in diverse dimensions of quality of life (QoL),^7^, ^8^, ^9^ with attacks limiting activities of daily living, decreasing productivity at work or school, and restricting social engagement.^10^ The overarching goals of management are to reduce or prevent attacks, improve the quality of healthcare experience, and minimize morbidity and mortality,^11^ and patient-centered approaches have been shown to improve adherence and outcomes.^12^, ^13^, ^14^ Despite advancements in therapeutic development, the patient experience and quality of care for individuals with HAE remain suboptimal. Given the complexity and chronicity of this disease, enhancing patient-centered care and service delivery has become a critical priority.^15^

Previous research has focused primarily on clinical management and therapeutic efficacy, while limited attention has been directed toward the patient's perspective and their experience throughout the continuum of care. Patient journey maps (PJMs) have emerged as a valuable tool in healthcare to refine service provision and better meet patient needs.^16^ By identifying key stages, barriers, and emotional dynamics, PJMs help clinicians and policy-makers design targeted interventions to improve outcomes and satisfaction.

This study aims to develop a HAE Patient Journey Map (HAE-PJM) tailored to the Chinese healthcare context. This mixed-methods, multi-step process comprising patient surveys, qualitative interviews with thematic analysis, and expert validation. Direct patient engagement allowed us to delineate core stages of the HAE journey, from symptom onset to long-term management. The findings inform strategies to enhance care coordination and patient experience.

## Methods

### Study design and reporting

We conducted a single-center, mixed-methods study integrating qualitative interviews with quantitative patient-reported outcomes to build and validate an HAE-PJM. The qualitative component was reported in line with Consolidated Criteria for Reporting Qualitative Research (COREQ), and the mixed-methods procedures were conducted in accordance with Good Reporting of A Mixed Methods Study (GRAMMS). Details of the structured interviews are described in the "Structured interviews" section.

### Setting and study population

Fifteen adults with HAE who were under follow-up at the Allergy Department of Tongji Hospital were recruited for the interviews between July 15, 2024 and November 30, 2024. The inclusion criteria were adults (≥18 years) with confirmed HAE (type 1/2 or nC1INH), diagnosed according to international criteria, who were probands and had received at least 1 HAE-specific therapy (icatibant or lanadelumab), which are currently the first-line on-demand and long-term prophylactic treatments for HAE in mainland China. Participants were required to be probands (the first individuals in their family to be diagnosed with HAE) in order to ensure that the reported patient journey reflected the initial, unguided path to diagnosis, thereby allowing for a clear identification of systemic diagnostic barriers and delays without the influence of prior family knowledge. Exclusion criteria: acquired angioedema (AAE), mast cell-mediated angioedema responsive to antihistamines/corticosteroids, inability to provide consent or complete assessments, and severe comorbid conditions interfering with participation.^17^

### Ethics

This study adhered to the ethical standards of the Declaration of Helsinki and its amendments. The study was approved by the Independent Ethics Committee of Tongji Hospital, Tongji Medical College, Huazhong University of Science and Technology, Wuhan, China (No. 2024-87-1). All participants signed a written informed consent form before the interviews, after they had received information about the study.

### Structured interviews

We used a structured interview guide (48 items; Supplementary file 1) in this study, including selective (closed-ended), open-ended, and in-depth questions (designed to probe deeper into respondents' thoughts and experiences). We aimed to summarize demographics, pre-diagnosis symptom onset and help-seeking, diagnostic pathways, therapeutic relationships with healthcare professionals, initiation of HAE therapy, and experiences of living with treated HAE. Each interview with patients, caregivers, and healthcare professionals lasted 30–50 min, was audio-recorded and transcribed verbatim within 24 h.

The interview protocol was developed by a multidisciplinary team (allergist, methodologist, nurse specialist) through literature scoping and discussion, and pilot-tested on 2 volunteers (data not included) to refine wording and flow. To minimize bias, interviewers were trained to use neutral prompts and avoid leading questions.

### Co-design and validating the HAE-PJM

Drawing on the information from the in-depth interviews, a patient journey map was crafted to depict the various phases patients navigate, encapsulating their emotional responses, healthcare interactions, and overall experiences. The initial map was then shared with stakeholders, including 6 patients, 8 caregivers, and 2 immunology professionals. An online expert group discussion was held in March 2025 to agree on stage definitions, touchpoints and priorities for improvements. The discussion was recorded, and the consensus decisions shaped the final HAE-PJM.

### Outcome measures: emotional status and angioedema control

Anxiety and depression were assessed using the Hospital Anxiety and Depression Scale (HADS), a 14-item instrument with 2 seven-item subscales (HADS-A, HADS-D), each scored 0–21; higher scores indicate worse symptoms.^18^ Conventional thresholds: 0–7 normal, 8–10 borderline, 11–21 abnormal. HADS was administered pre-diagnosis (baseline), at diagnosis and post-treatment to evaluate emotional change. Disease control was assessed with the Angioedema Control Test (AECT), a validated 4-item patient-reported measure (total score 0–16, ≥10 as well-controlled).^19^ The AECT was administered post-treatment, referencing disease control over the recent weeks.

### Statistical analysis

This study included a quantitative component to evaluate pre–post changes in anxiety and depression and to provide quantitative support for identifying key touchpoints and improvement opportunities in the HAE-PJM. Statistical analyses were conducted according to a prespecified plan, used two-sided tests, and with α = 0.05. HADS scores were computed per the instrument manual; when ≤1 item was missing within a subscale, the missing item was imputed using the mean of the completed items for that subscale. Pre-post differences in HADS-A, HADS-D, and AECT scores were compared with paired t-tests, Wilcoxon signed-rank tests were used as sensitivity analyses. All analyses and graphics were performed in GraphPad Prism 8.0. The HAE-PJM figure was created with BioRender (https://app.biorender.com/).

## Results

### Characteristics of the study population

Fifteen patients suffering from HAE were recruited for our study. Two-thirds were female, and all had a disease duration of more than 5 years. The mean time from first symptom to confirmed diagnosis was 16.3 years, with 9/15 (60%) reporting a diagnostic delay exceeding 10 years (Table 1). Among them, 13 patients were diagnosed with HAE-C1INH-Type1, while the subtype remained unidentified for the remaining 2 patients due to unavailable historical laboratory records. Laboratory profiles, including Complement C4 level, C1INH antigen level, and C1INH functional activity for each patient, are detailed in Supplementary Table 1.

**Table 1:** Demographic and clinical characteristics of the study population.

Characteristic	Patients with HAE (n = 15)
Age (years), mean ± SD	37.20 ± 10.35
Gender, N (%)	
Male	5 (33.33)
Female	10 (66.67)
Education, N (%)	
Below college	8 (53.33)
Bachelor	4 (26.67)
Postgraduate	3 (20.00)
Marriage and bearing,N (%)	11 (73.33)
Employed, N (%)	12 (80.00)
Household average income monthly, N (%)	
≤5000 yuan	5 (33.33)
5000–10000 yuan	6 (40.00)
≥10000 yuan	4 (26.67)
Medical insurance, N (%)	15 (100.00)
Family history, N (%)	12 (80.00)
Age of onset (years), mean ± SD	17.77 ± 10.81
Age at diagnosis (years), mean ± SD	34.07 ± 9.61
Time from symptom onset to diagnosis, (years), mean ± SD	16.30 ± 12.34
Within 5 years, N (%)	1 (6.67)
Within 5–10 years, N (%)	5 (33.33)
>10 years, N (%)	9 (60.00)

HAE, Hereditary Angioedema; N, Number; SD, Standard Deviation

### Pre-diagnosis experience and unmet needs

#### Symptoms and first presentations

**Fig. 1:**
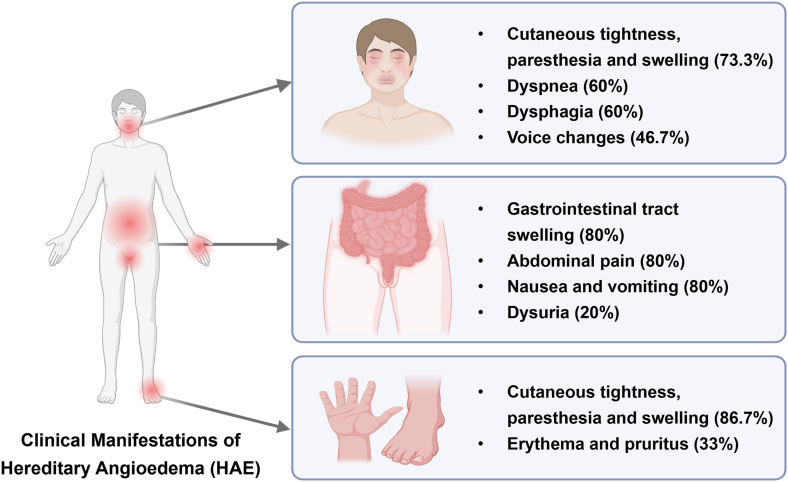
**Clinical Manifestations of Hereditary Angioedema (HAE).** Patients with HAE present with heterogeneous symptoms involving multiple systems. Head and neck manifestations included cutaneous tightness, swelling (73.3%), dyspnea (60%), dysphagia (60%), and voice changes (46.7%). Gastrointestinal involvement was frequent (80%), with abdominal pain, nausea/vomiting, and dysuria (20%). Cutaneous swelling of the extremities occurred in 86.7% of patients, often with erythema and pruritus (33%). Percentages indicate proportions in our cohort (n = 15)

At symptom onset, 12/15 (80%) patients experienced prodromal symptoms, most frequently cutaneous tightness or paresthesia (11/15, 73.3%), erythema or pruritus (5/15, 33.3%), and nausea (4/15, 26.7%). The first HAE manifestation was typically extremity edema (13/15, 86.67%), while 2/15 (13.3%) reported gastrointestinal (GI) edema as their first symptom. During progression, multisite involvement of edema was common (12/15, 80%), with laryngeal edema in 10/15 (66.7%) representing a life-threatening emergency. Gastrointestinal involvement (12/15, 80.0%) and perineal involvement (7/15, 46.7%) were frequently misdiagnosed as gastroenteritis and gynecological disorders (Fig. 1). Percentages indicate proportions in our cohort (n = 15).

**Fig. 2:**
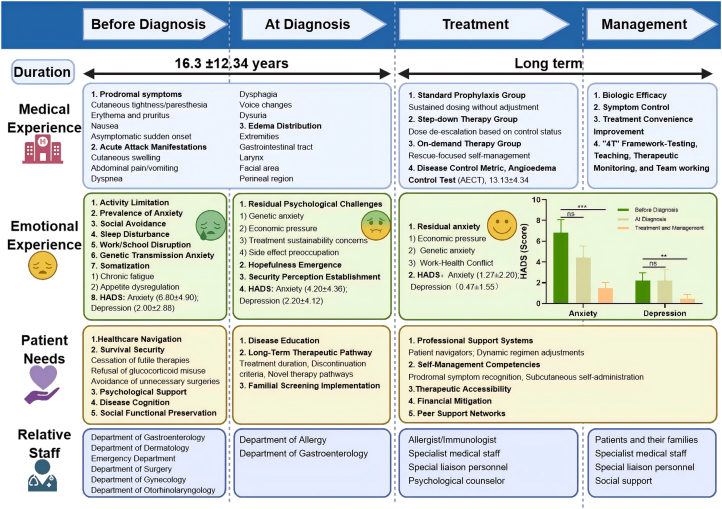
**Hereditary angioedema patient journey map (HAE-PJM) across disease phases**. This map summarizes the HAE care continuum in China, spanning pre-diagnosis, at diagnosis, treatment, and long-term management. It integrates medical features, emotional burden (HADS), disease control (AECT), patient needs, and roles of healthcare staff. Findings highlight delayed diagnosis, persistent psychosocial stress, and systemic barriers to treatment access, while outlining opportunities for multidisciplinary collaboration, education, and patient navigation to optimize patient-centered care

#### Disease burden and quality of life impact before diagnosis

Prior to diagnosis, uncontrolled HAE imposed a significant multidimensional burden on patient health and daily living. Patients reported extensive impairment in QoL, impairment across 3 core domains (Fig. 2).

In the functional domain, limitations in physical activity (73.3%) and interruptions in work or studies (66.7%) were most frequently reported, severely affecting patients' ability to fulfill social and occupational roles. On the psychological level, a high degree of generalized anxiety (66.7%) and social avoidance (53.3%) due to fear of public attacks, which was further quantified by an elevated baseline HADS-A score (6.80 ± 4.90). Additionally, somatic symptoms such as sleep disturbances (46.7%), chronic fatigue (26.7%), and appetite dysregulation (33.3%), which collectively depleted patients’ energy and well-being.

#### Healthcare utilization and misdiagnosis

Patients sought care across multiple specialties, including gastroenterology (8/15, 53.3%), dermatology (7/15,46.7%), emergency medicine (7/15, 46.7%), surgery (5/15, 33.3%), gynecology (3/15, 20.0%), otolaryngology (2/15, 13.3%) and rheumatology (2/15,13.3%). Misdiagnosis occurred in 12/15 (80.0%), including allergy, urticaria, gastroenteritis, peritonitis, pelvic infection, pancreatitis, ascites, and pelvic effusion. These led to inappropriate treatments (antihistamines, antibiotics, glucocorticoids, and occasional surgery). Without a clear diagnosis, respondents generally felt anxious (10/15, 66.7%) and frequent social avoidance (8/15,53.3%).

#### Unmet needs prior to diagnosis

Five major domains emerged during respondents' pre-diagnosis period: (i) First, healthcare navigation needs (15/15, 100%)—lack of timely diagnostic pathways and specialized screening; (ii) survival/security (13/15, 86.7%) experienced futile therapies, (10/15, 66.7%) reported prior glucocorticoid misuse with no clinical benefit, and (2/15, 13.3%) had undergone unnecessary surgeries; (iii) psychological support needs—reflected the profound anxiety and distress caused by diagnostic delays and misdiagnoses; (iv) disease literacy 14/15 (93.3%)—desire to understand clinical features and inheritance; and (v) social functional preservation needs affected 11/15 (73.3%) of respondents, who struggled to maintain work, education, and family responsibilities during prolonged diagnostic odysseys. These results demonstrate significant systemic deficiencies in the early recognition of hereditary angioedema (HAE), emphasizing the critical need for enhanced clinician education regarding diagnostic red flags, empowered patient advocacy to facilitate access to appropriate therapeutic interventions, and implementation of comprehensive support services that concurrently address the medical and psychosocial challenges encountered.

### Emotional experience and patient needs at diagnosis

#### Definitive diagnosis

Most diagnoses were established in the allergy department for (13/15, 86.7%), with the remainder in the gastroenterology department (2/15, 13.3%). Compared with baseline, anxiety decreased (HADS-A: 6.80 ± 4.90 to 4.20 ± 4.36, p > 0.05), while depression remained stable (HADS-D: 2.00 ± 2.28 to 2.20 ± 4.12, p > 0.05), with no significant change observed. Despite this, 13/15 (86.7%) exhibited persistent psychological challenges, predominantly fear of passing the disease to offspring (11/15,73.3% at-diagnosis vs. 4/15, 26.7% pre-diagnosis), economic stress (9/15, 60.0%), worries about treatment sustainability (8/15, 53.3%), and fear of adverse effects (2/15, 13.3%). Notably, positive psychological improvements were observed: 12/15 (80.0%) of patients felt increased hopefulness, while 11/15 (73.3%) described attaining a meaningful sense of security.

#### Disease awareness before and at diagnosis

At diagnosis phase, all patients with HAE emphasized the need for comprehensive disease education, including disease mechanisms, symptom management, and triggers. Notably, 7/15 (46.7%) patients reported no prior awareness of the disease, while 5/15 (33.3%) learned via symptom-driven searches or social media platforms, prompting them to seek medical evaluation; and 4/15 (26.7%) patients gained limited disease awareness through physician-initiated HAE screening. These findings underscore significant gaps in disease recognition prior to formal diagnosis, highlighting the critical role of both healthcare provider engagement and responsible health information dissemination. Additionally, all patients 15/15 (100%) requested comprehensive education and a clearly defined long-term therapeutic pathway, encompassing treatment duration, discontinuation criteria, and access to novel therapies. However, only 7/15 (46.7%) expressed interest in family screening, indicating a significant gap in the systematic cascade detection of high-risk relatives.

### Treatment and management

#### Disease control

During the management phase,12/15 (80.0%) reported reduced attack frequency and longer attack-free periods. When breakthrough attacks occurred, patients noted milder symptoms and shorter duration compared to pretreatment baseline. These subjective reports were corroborated by quantitative measures, with an overall Angioedema Control Test (AECT). The mean AECT score was 13.13 ± 4.34. Three distinct treatment strategies were implemented based on careful clinical and socioeconomic considerations: (i) Standard Prophylaxis, 6/15 (40%) maintained on fixed-interval lanadelumab (2–4 weeks per injection) achieved optimal control (AECT: 14.67 ± 1.97). (ii) Step-down Therapy, 6/15 (40%) demonstrated good control (AECT: 13.33 ± 5.09), though 1 non-adherence patient reported poor control (AECT = 3), showing suboptimal outcomes due to financial constraints. (iii) On-demand Therapy, 3/15 (20%) using icatibant showed variable control (AECT: 9.67 ± 5.69), with treatment choices primarily driven by economic factors (n = 2) or safety concerns (n = 1). Notably, 9/15 (60%) of participants reported treatment access barriers, including: procurement complexity, reimbursement difficulties, storage requirements, and injection administration. These findings highlight both the clinical effectiveness of current HAE therapies and the persistent systemic barriers limiting optimal management.

#### Psychosocial changes from baseline after treatment

Following treatment, HADS scores improved significantly indicating improved psychological well-being. Quantitative assessment using the HADS demonstrated low scores compared to pre-diagnosis levels for both anxiety (HADS-A, 1.27 ± 2.20, p < 0.001) and depression (HADS-D, 0.47 ± 1.55, p < 0.01), indicating overall good mental health status. Nevertheless, 9/15 (60.0%) patients still experienced residual anxiety, (i) economic pressure (8/15, 53.3%), characterized by persistent worries about treatment costs, insurance coverage, and long-term financial burden of disease management; (ii) fear of passing the disease to offspring (5/15,33.3%), reflecting concerns regarding disease transmission to offspring and family planning implications; and (iii) work-health conflict (5/15,33.3%), representing challenges in balancing professional responsibilities with disease management needs.

#### Patient needs in HAE management

In the treatment and management phase, HAE patients had 5 key needs. All of them emphasized the professional support systems, including 10/15 (66.7%) of them need patient navigators and 9/15 (60.0%) need dynamic regimen adjustments. Self-management competencies were highlighted by 13/15 (86.7%) of patients, particularly prodromal symptom recognition and subcutaneous self-administration. Additionally, therapeutic accessibility was a concern for 9/15 (60.0%) of participants, while financial mitigation (8/15, 53.3%) and peer support networks (5/15, 33.3%) were also identified as significant challenges.

### Expert-patient group validation of the HAE-PJM

The expert-patient group validated the HAE-PJM and emphasized role-specific responsibilities across the diagnostic and treatment phases, as summarized in Table 2. Participants emphasized patient self-management, including trigger identification and maintenance of attack diaries. Physicians were recognized for their role during diagnosis, risk stratification, and individualized therapy, and highlighted the contributions of nurses to patient education and triage. Patient navigators were valued for ensuring continuity of care and logistical coordination, while societal/health-system support was deemed essential for public awareness and structural accommodations. Collectively, the expert group confirmed that this multidisciplinary configuration facilitates earlier recognition, improves treatment adherence, and strengthen overall disease management, in alignment with the HAE-PJM framework.

**Table 2:** Roles and responsibilities in HAE comprehensive management

Role	Primary Responsibility	Diagnostic Phase	Treatment & Follow-up Phase
**Patient**	Self-management and early recognition	• Learn HAE characteristics and triggers • Keep an attack diary (timing, location, triggers, severity) • Seek psychological and social support	• Carry medical identification and emergency action plan • Maintain and self-administer on-demand therapy c Communicate family history and screening among relatives
**Physician**	Diagnosis, individualized therapy, and care coordination	• Maintain suspicion for recurrent angioedema or abdominal pain and consider HAE promptly • Confirm C4,C1–INH assays, genetic testing • Perform risk stratification	• Prescribe biologic therapies as appropriate • Provide emergency action plan for acute attacks • Facilitate family genetic counseling and screening • Coordinate with multidisciplinary partners
**Nurse**	Patient education, monitoring, and initial triage	• Avoidance of triggers and early symptom recognition • Triage acute symptoms and activate rapid response pathways for severe attacks	• Teach correct medication use and self-injection technique • Monitor adherence and adverse events during follow-up
**Patient navigator**	System navigation and continuity of care	• Provide simplified educational materials and link patients with support organizations	• Coordinate clinic visits, diagnostic workups, and follow-ups • Assist with access to medications, insurance, and financial support • Remind patients of scheduled appointments and collect feedback • Establish post-crisis communication pathways
**Societal support**	Structural support and awareness raising	• Promote early recognition through public education and primary care/provider training • Develop and disseminate acute management protocols	• Ensure accessibility and reimbursement for prophylactic therapies • Establish and support rare disease registries and related research • Advocate for workplace accommodations and anti-discrimination protections

The phased structure of the HEAPJM was also confirmed, with a strong call for clear communication pathways and accessible resources across both diagnostic and treatment management phases. Integration of psychological support, systematic family screening, and written emergency action plans received particular endorsement, reflecting the model's holistic orientation. Stakeholder feedback informed minor refinements to stage definitions and role interfaces, reinforcing the HAE-PJM's utility as a practical guide for coordinated HAE care.

## Discussion

Our study represents the first systematic effort to establish the HAE-PJM in mainland China. By integrating qualitative interviews with quantitative patient-reported outcomes and validating findings with multiple stakeholders, we provided a comprehensive understanding of the diagnostic, psychosocial, and systemic barriers faced by Chinese patients. This mixed-methods approach not only highlights gaps in early recognition, psychosocial support, and treatment access but also generates actionable insights to guide healthcare system reform and patient-centered care strategies.

Previous studies have explored aspects of the HAE^20^, ^21^, ^22^ and its patient journey from different perspectives. These have comprised expert consensus approaches, including Banerji et al^23^ and Caballero et al^24^ without patient-level data; investigations into the psychological impact of prophylaxis,^25^ and qualitative explorations of patient experiences in specific populations, such as the pioneering work by Vargas Camaño et al in Mexico.^26^ Building upon these efforts, our study contributes to this field by integrating qualitative narratives with quantitative patient-reported outcomes to develop and validate the first systematic and stakeholder-validated HAE-PJM in China. This integration has yielded a more robust understanding of barriers and opportunities across the continuum of HAE care.

Our findings underscore 3 major gaps. First, by focusing on probands, our data revealed prolonged diagnostic delays, far exceeding the global benchmark of 8–10 years.^27^ Misdiagnosis, inappropriate glucocorticoid use, and even surgery highlight underuse of C1INH testing and limited referral pathways, consistent with international reports.^28^, ^29^, ^30^ Second, psychological support are insufficiently addressed. Concerns about disease inheritance, economic stress, and work–health conflicts persisted despite therapy, showing that HAE management cannot rely solely on pharmacological control. Embedding genetic counseling, psychological support, and financial navigation into the care pathway is essential, corroborating previous evidence from China.^31^^,^^32^ Third, system-level barriers constrain treatment effectiveness. Although therapies such as lanadelumab and icatibant are efficacious, real-world use is limited by reimbursement, procurement, and self-administration challenges.^33^ Multidisciplinary models, including patient navigators and digital tools, may provide practical solutions, as supported by the successful implementation of hub-and-spoke networks in other regions.^34^ The “4A” framework—Awareness, Access, Advocacy, and Alliance—advanced by Li et al^35^ highlights progress in collaborative “Hub-and-Spoke” networks, while Wong et al.^36^ showed that mislabeled drug allergies—present in 22% of HAE patients—can prolong diagnostic delays and worsen outcomes, but are reversible after testing.

From our findings, we propose the “4T″ framework (Testing, Teaching, Therapeutic Monitoring, Team working) as a proposed care model. It should be emphasized that this is a data-driven conceptual synthesis requiring future validation and is not a tested intervention from this study. (i) Testing—Healthcare workers establish red-flag triggers for testing (recurrent non-urticarial angioedema, C4 levels, quantitative/functional C1INH, and genetic testing). (ii) Teaching—Healthcare workers deliver structured education at diagnosis, provide written emergency action plans, and train self-injection when applicable. (iii) Therapeutic Monitoring—Track disease control with AECT (0–16; ≥10 as well-controlled) and mental health with HADS at predefined intervals; adjust regimens using shared decision-making; address adherence and adverse events. (iv) Team working—Operate allergist-led MDTs.^37^, ^38^, ^39^ This framework translates patient-identified barriers into strategies for improving diagnosis, education, monitoring, and multidisciplinary care.^40^^,^^41^

As the first HAE-PJM in China, our study still has several limitations. The probands were recruited from families across various regions of China, and the sample size followed the principle of thematic saturation—a common standard in qualitative rare disease research. However, the modest sample size (n = 15) and the single-center design may still affect the statistical robustness of the findings. These factors could also limit the generalizability of the results. Furthermore, the cross-sectional nature of our data precludes longitudinal assessment of the patient journey.

Future work will incorporate more quantitative measures, including disease-specific quality-of-life instruments, to evaluate the impact of interventions. Building directly on the specific barriers identified here, we are currently exploring their prevalence and impact in a larger, multi-center cohort. This represents a critical next step toward validating the cost-effectiveness and scalability of our proposed solutions, including patient navigation and digital health tools within the 4T framework, to advance HAE care toward a patient-centered, multidisciplinary model in China.

## Conclusion

This study establishes China's first stakeholder-validated HAE-PJM, integrating qualitative and quantitative data to identify critical gaps during diagnosis, care coordination, and treatment implementation. Three priority actions are proposed: standardized diagnostic pathways, integrated psychosocial and self-management support, and access-oriented care models. While limited by its single-center design, this work provides a foundational framework for optimizing HAE care. Future efforts should focus on multicenter validation, implementation science, and digital health tools to enable scalable, patient-centered care aligned with the “4T” framework (Testing, Teaching, Therapeutic Monitoring, and Team working), ultimately bridging the gap between efficacy and real-world outcomes.

## Availability of data and materials

The datasets generated and/or analyzed during the current study are not publicly available due to patient privacy and ethical restrictions but are available from the corresponding author on reasonable request and with approval from the Ethics Committee of Tongji Hospital, Tongji Medical College, Huazhong University of Science and Technology, Wuhan, China.

## Author contributions

**Yin Wang, MM**: conceptualization; methodology; patient recruitment and data collection; drafting of the manuscript. **Yangxue Fu, PhD**: methodology; data analysis and interpretation; visualization; critical revision of the manuscript. **Hao Chen, PhD and Yaqi Yang, MD**: patient recruitment; clinical data acquisition; support in interview transcription. **Jin Liu, MD and Qingxiu Xu, MD:** statistical analysis; assistance in quantitative outcomes evaluation (HADS, AECT). **Si Zhang, BS:** patient management, data curation, and collection of clinical records and audio materials. **Jing Cheng, MD:** validation; supervision; provided critical feedback on study design and manuscript. **Rongfei Zhu, MD, PhD:** project administration; overall supervision; final approval of the version to be published.

## Ethics approval

This study adhered to the ethical standards of the Declaration of Helsinki and its amendments. The study was approved the independent Ethics Committee of Tongji Hospital, Tongji Medical College, Huazhong University of Science and Technology, Wuhan, China (No. 2024-87-1).

## Declaration of Generative AI and AI-assisted technologies in the writing process

No generative AI tools were used in the writing, editing, or figure preparation of this manuscript.

## Funding

Funding for this study was provided by the Scientific Research Fund of 10.13039/501100015640Tongji Hospital (Grant No. 2025C10).

## Declaration of competing interest

The authors declare that they have no competing interests.
