# Supplementary material for: Developing a patient journey map to improve care and experience in Chinese patients with hereditary angioedema
Source: World Allergy Organ J. 2026 Jan 30;19(2):101333. doi: 10.1016/j.waojou.2026.101333 (PMC12878669; doi:10.1016/j.waojou.2026.101333)
Supplement: Multimedia component 1 [file mmc1.docx]

**Supplementary Table 1. Individual Patient Data for Confirmation of Hereditary Angioedema (HAE) Diagnosis**

| Interview ID | Gender | Age (years) | Age at Onset (years) | Age at Diagnosis (years) | C4 Level  (ug/ml) | C4 Normal range (ug/ml) | C1INH Level  (ug/ml) | C1INH  Normal range  (ug/ml) | C1INH Functional Activity  （≥58.9%） | Type |
| --- | --- | --- | --- | --- | --- | --- | --- | --- | --- | --- |
| 1 | F | 26 | 12 | 26 | 34.87 | 72.85-372.95 | 23.49 | 81.46-291.29 | <7% | HAE-C1INH-Type1 |
| 2 | F | 32 | 10 | 31 | 18.91 | 72.85-372.95 | 11.00 | 81.46-291.29 | <7% | HAE-C1INH-Type1 |
| 3 | F | 30 | 19 | 28 | 60.00 | 160.0-380.0 | 70.00 | 81.46-291.29 | - | HAE-C1INH-Type1 |
| 4 | F | 32 | 18 | 31 | 43.27 | 72.85-372.95 | 17.73 | 81.46-291.29 | <7% | HAE-C1INH-Type1 |
| 5 | F | 24 | 18 | 24 | 18.81 | 72.85-372.95 | 11.81 | 81.46-291.29 | <7% | HAE-C1INH-Type1 |
| 6 | F | 40 | 19 | 30 | 29.49 | 72.85-372.95 | 6.48 | 81.46-291.29 | <7% | HAE-C1INH-Type1 |
| 7 | F | 48 | 0.5 | 47 | 149.12 | 72.85-372.95 | 51.20 | 81.46-291.29 | <7% | HAE-C1INH-Type1 |
| 8 | M | 31 | 23 | 31 | 155.27 | 72.85-372.95 | 51.11 | 81.46-291.29 | 18.63% | HAE-C1INH-Type1 |
| 9 | M | 39 | 10 | 26 | 50.00 | 100.0-400.0 | 50.00 | 210.0-390.0 | 14% | HAE-C1INH-Type1 |
| 10 | M | 39 | 20 | 39 | 46.49 | 72.85-372.95 | 32.79 | 81.46-291.29 | <7% | HAE-C1INH-Type1 |
| 11 | F | 30 | 26 | 28 | 23.26 | 72.85-372.95 | 12.55 | 81.46-291.29 | <7% | HAE-C1INH-Type1 |
| 12 | F | 56 | 20 | 50 | - | - | - | - | - | Unidentified |
| 13 | F | 28 | 18 | 26 | 20.00 | 160.0-380.0 | - | - | - | Unidentified |
| 14 | M | 48 | 5 | 40 | 43.73 | 72.85-372.95 | 14.12 | 81.46-291.29 | <7% | HAE-C1INH-Type1 |
| 15 | M | 55 | 48 | 54 | <6.25 | 72.85-372.95 | <6.25 | 81.46-291.29 | <7% | HAE-C1INH-Type1 |

ID, Identification; C4, Complement 4; C1INH, C1 Inhibitor; ug/mL, Micrograms per milliliter.
