# Supplementary material for: Developing a patient journey map to improve care and experience in Chinese patients with hereditary angioedema
Source: World Allergy Organ J. 2026 Jan 30;19(2):101333. doi: 10.1016/j.waojou.2026.101333 (PMC12878669; doi:10.1016/j.waojou.2026.101333)
Supplement: Multimedia component 2 [file mmc2.docx]

**Supplementary Table 2. Therapeutic options for hereditary angioedema in mainland China.**

| **Treatment Phase** | **Drug Type** | **Drug** | **Brand Name** | **Recommendation Level** | **Availability in China** |
| --- | --- | --- | --- | --- | --- |
| **Acute Phase Treatment** | Bradykinin B2 receptor antagonist | Icatibant | Fyzenyo | First-line | Available |
|  | Intravenous C1INH replacement therapy | Plasma-derived C1INH | Berinert | First-line | Not yet approved |
|  |  |  | Cinryze | First-line | Not yet approved |
|  |  | Domestic C1 esterase inhibitor | - | - | Phase III clinical trial |
|  |  | Recombinant C1INH | Ruconest | First-line | Not yet approved |
|  | Plasma kallikrein inhibitor | Ecallantide | Kalbitor | First-line | Not yet approved |
|  | Fresh frozen plasma (FFP) | - | - | Second-line | Available |
| **Short-term Prophylaxis** | Weak androgens | Danazol | - | Second-line | Commonly used |
|  | Antifibrinolytic agent | Tranexamic acid | - | Second-line | Available |
|  | Intravenous C1INH replacement therapy | Plasma-derived C1INH | Berinert | First-line | Not yet approved |
|  |  |  | Cinryze | First-line | Not yet approved |
| **Long-term Prophylaxis** | Anti-plasma kallikrein monoclonal antibody | Lanadelumab | Takhzyro | First-line | Available |
|  | Weak androgens | Danazol | - | Second-line | Commonly used |
|  | Antifibrinolytic agent | Tranexamic acid | - | Second-line | Available |
|  | Intravenous C1INH replacement therapy | Plasma-derived C1INH | Haegarda | First-line | Not yet approved |
|  |  |  | Cinryze | First-line | Not yet approved |
|  | Plasma kallikrein inhibitor | Berotralstat | - | First-line | Not yet approved |
|  | Anti-factor XIIa monoclonal antibody | Garadacimab | - | - | Not yet approved |
