# Supplementary material for: Developing a patient journey map to improve care and experience in Chinese patients with hereditary angioedema
Source: World Allergy Organ J. 2026 Jan 30;19(2):101333. doi: 10.1016/j.waojou.2026.101333 (PMC12878669; doi:10.1016/j.waojou.2026.101333)
Supplement: Multimedia component 3 [file mmc3.docx]

**Patient Interview**

**PERSONAL DETAILS**

1. Name：
2. Sex:
3. Age：
4. Education level: high school/college/ undergraduate/postgraduate
5. Marital status: unmarried/married
6. Fertility status: no children/have children
7. Are you employed?

If answer = Yes: full-time, part-time, full-time student

If answer = No: retired, not able to work/disabled, currently not employed

1. Household average income monthly:

5000 yuan or below/5000-10000 yuan/ more than 10000 yuan

1. Medical care assurance: self-funded/medical insurance/free medical service

**BEFORE DIAGNOSIS**

The following questions refer to your life before the diagnosis. So, we need you to remember what your life was like before diagnosis, but when you already have hereditary angioedema symptoms.

1. Age at onset:
2. How often did you experience these symptoms?(Frequency of attacks)

very frequently/frequently/occasionally/rarely

12. Treatment drugs for the attack:

13. Past Medical history:

14. Family history:

15. Which of the following factors have ever induced your oedema attacks:

Emotional stress (high or low)

Changes in hormonal levels (e. g. before and after menstruation)

Trauma

Exercise (what kind of exercise, how long)

Dental treatment

Surgery, medical procedures

Infection or other diseases

Using drugs (which drugs are indicated)

Weather change (which weather change)

Food (which food)

Drinking alcohol

Lack of sleep

Fatigue

Constipation

Other factors:

Without obvious inducement

16. Whether you have had the following experiences before the onset of oedema:

Skin erythema and itching

Temper

Hunger

Tired

Nausea

Skin tightness or tingling sensation

Noise

Other experiences:

No discomfort before any occurrence

17. Which of the following areas develop oedema during oedema onset:

Limbs

Face part: eyelids, face, lips

Throat: pharynx, mouth, throat, larynx, tongue

Trunk: abdomen, back, buttocks

Gastrointestinal tract: obvious abdominal pain

Perineal: groin, genitalia, bladder

18. What symptoms occur when edema occurs?

Skin swelling

Abdominal pain

Nausea

Vomiting

Diarrhea

Constipation

Vertigo

Headache

Tired

Dyspnea

Dysphagiaand

Sound changes

Dysuria

Other symptoms:

19.Impact on quality of life before diagnosis:

|  | **Yes** | **No** |
| --- | --- | --- |
| Anxiety,fear,and depression |  |  |
| Affect your study and work |  |  |
| Worried about inheriting to their children |  |  |
| Affect the daily activities |  |  |
| Influence tourism |  |  |
| Affect social activities |  |  |
| Affect appetite |  |  |
| Often feel tired |  |  |
| Affect sleep quality |  |  |

20. Hospital Anxiety and Depression Scale，HADS

Tick the box beside the reply that is closest to how you have been feeling before diagnosis. Don’t take too long over you replies: your immediate is best.

| **D** | **A** |  | **D** | **A** |  |
| --- | --- | --- | --- | --- | --- |
|  |  | **I feel tense or 'wound up':** |  |  | **I feel as if I am slowed down:** |
|  | 3 | Most of the time | 3 |  | Nearly all the time |
|  | 2 | A lot of the time | 2 |  | Very often |
|  | 1 | From time to time, occasionally | 1 |  | Sometimes |
|  | 0 | Not at all | 0 |  | Not at all |
|  |  |  |  |  |  |
|  |  | **I still enjoy the things I used to enjoy:** |  |  | **I get a sort of frightened feeling like 'butterflies' in the stomach:** |
| 0 |  | Definitely as much |  | 0 | Not at all |
| 1 |  | Not quite so much |  | 1 | Occasionally |
| 2 |  | Only a little |  | 2 | Quite Often |
| 3 |  | Hardly at all |  | 3 | Very Often |
|  |  |  |  |  |  |
|  |  | **I get a sort of frightened feeling as if**  **something awful is about to happen:** |  |  | **I have lost interest in my appearance:** |
|  | 3 | Very definitely and quite badly | 3 |  | Definitely |
|  | 2 | Yes, but not too badly | 2 |  | I don't take as much care as I should |
|  | 1 | A little, but it doesn't worry me | 1 |  | I may not take quite as much care |
|  | 0 | Not at all | 0 |  | I take just as much care as ever |
|  |  |  |  |  |  |
|  |  | **I can laugh and see the funny side of things:** |  |  | **I feel restless as I have to be on the move:** |
| 0 |  | As much as I always could |  | 3 | Very much indeed |
| 1 |  | Not quite so much now |  | 2 | Quite a lot |
| 2 |  | Definitely not so much now |  | 1 | Not very much |
| 3 |  | Not at all |  | 0 | Not at all |
|  |  | **Worrying thoughts go through my mind:** |  |  | **I look forward with enjoyment to things:** |
|  | 3 | A great deal of the time | 0 |  | As much as I ever did |
|  | 2 | A lot of the time | 1 |  | Rather less than I used to |
|  | 1 | From time to time, but not too often | 2 |  | Definitely less than I used to |
|  | 0 | Only occasionally | 3 |  | Hardly at all |
|  |  |  |  |  |  |
|  |  | **I feel cheerful:** |  |  | **I get sudden feelings of panic:** |
| 3 |  | Not at all |  | 3 | Very often indeed |
| 2 |  | Not often |  | 2 | Quite often |
| 1 |  | Sometimes |  | 1 | Not very often |
| 0 |  | Most of the time |  | 0 | Not at all |
|  |  |  |  |  |  |
|  |  | **I can sit at ease and feel relaxed:** |  |  | **I can enjoy a good book or radio or TV program:** |
|  | 0 | Definitely | 0 |  | Often |
|  | 1 | Usually | 1 |  | Sometimes |
|  | 2 | Not Often | 2 |  | Not often |
|  | 3 | Not at all | 3 |  | Very seldom |

Scoring:

Total score: Depression (D) Anxiety (A)

0-7= Normal

8-10=Borderline abnormal (borderline case)

11-21= Abnormal (case)

**DIAGNOSIS**

21.When were you diagnosed with HAE**?**

Month： Year： (Your age at this time: )

22. How did the diagnosis affect you? What were you thinking about?

23. When your symptoms first started, what healthcare providers did you consult before finally being diagnosed (e.g.general practitioner, emergency physician, physician, surgeon...)?

24. Do you remember how many visits were diagnosed with HAE? Number of visits:

25. Were there any misdiagnoses? If yes, which ones?

26. Did you also receive incorrect treatments due to these misdiagnoses? if yes, which ones?

27. Which physician/healthcare provider actually made the final diagnosis?

28. Did you know about HAE before being diagnosed?

29. Did you feel your healthcare professionals spend enough time to discuss your diagnosis and address your concerns?

30. Were you satisfied with the information you received from your healthcare professional?(Yes / No, if not, please explain why you were dissatisfied.）

31. How did you feel after after being diagnosed?(For example, hopeful, lost, alone, supported...）

32. What would have helped to make you feel better?

33. Impact on quality of life after diagnosis(specific items refer to question 19):

34. HADS Total score(specific items refer to question 20):

Depression (D) Anxiety (A)

**TREATMENT**

1. Who is/was your attending healthcare provider after the diagnosis? (e.g.general practitioner, physician,emergency physician, allergy physician,Other: )

36. Please think back to the time when you were diagnosed and possibly received a certain medication and/or treatment:

a) How did you feel at that time?

b) Was there anything that particularly helped you to cope better with HAE?

37. What treatment did you receive/are you receiving? (Please list chronologically all therapies received)

38. Does or did the treatment have an impact on your life/your everyday life? (Family,

partnership, job, interests and hobbies...)?

Yes / No (If Yes: please describe the effect of the treatment.）

**LIVING WITH HAE**

39.How often do you have a further consultation?

a) What is done during these further consultations?

40. Angioedema Control Test (AECT)Instructions and Scoring

|  | 0 Points | 1 Point | 2points | 3points | 4 Points |
| --- | --- | --- | --- | --- | --- |
| In the last 4 weeks, how often have you had angioedema? | very often | often | sometimes | seldom | not at all |
| In the last 4 weeks, how much has your quality of life been affected by angioedema? | very much | much | somewhat | a little | not at all |
| In the last 4 weeks, how much has the unpredictability of your angioedema bothered you? | very much | much | somewhat | a little | not at all |
| In the last 4 weeks, how well has your angioedema been controlled by your therapy? | not at all | a little | somewhat | well | very well |

**Total score:**

AECT score <10 points indicates poorly controlled recurrent angioedema,AECT score ≥10 points indicates well-controlled recurrent angioedema.

41.Have your symptoms ever changed over time?

Yes / No; if yes: please describe how they have changed?

42.Impact on quality of life after treatment(specific items refer to question 19):

43.What challenges does HAE pose in everyday life?

Family: Partner: Job: Interests and hobbies: Mental health:...

44.When it was clear that you had HAE:Did you look for further information about it?

Yes / No; if yes, where?On the internet/ Self-help groups / Patient Organizations/ my physician/in patient brochures provided to me by hospital/ Other:

1. Looking back, what do you wish had been different regarding your experience with HAE?
2. What would have helped you? (e.g. special contacts, special services)
3. Do you have any specific coping strategies to manage your Cervical Dystonia symptoms? yes / no; If yes, which ones?
4. How do you feel about your disease today?
5. What would you like to achieve for yourself?What time?
6. HADS Total score(specific items refer to question 20):

Depression (D) Anxiety (A)
